# A new karyotype for the spiny rat *Clyomys laticeps* (Thomas, 1909) (Rodentia, Echimyidae) from Central Brazil

**DOI:** 10.3897/CompCytogen.v6i2.1980

**Published:** 2012-04-09

**Authors:** Alexandra M. R. Bezerra, Juliana M. Pagnozzi, Ana Paula Carmignotto, Yatiyo Yonenaga-Yassuda, Flávio H. G. Rodrigues

**Affiliations:** 1 Departamento de Zoologia, Universidade de Brasília, Campus Darcy Ribeiro, Asa Norte, 70910-900, Brasília, DF, Brazil; 2 Faculdade Salesiana de Vitória, Av. Vitória 950, Forte São João, 29017-950, Vitória, ES, Brazil; 3 Universidade Federal de São Carlos, Campus Sorocaba, Rodovia João Leme dos Santos (SP-264), km 110, Bairro Itinga, 18052-780, Sorocaba, SP, Brazil; 4 Instituto de Biociências, Universidade de São Paulo, Rua do Matão 277, Cidade Universitária, 05508-090, São Paulo, SP, Brazil; 5 Departamento de Biologia Geral, Universidade Federal de Minas Gerais, Av. Antônio Carlos 6627, Pampulha, CP 486, 31270-901, Belo Horizonte, MG, Brazil; and Instituto Pró-Carnívoros.

**Keywords:** *Clyomys*, Cerrado, cytogenetic, Echimyidae, taxonomy, semifossorial habit

## Abstract

*Clyomys* Thomas, 1916 is a semifossorial rodent genus of spiny rats represented by only one species, *Clyomys laticeps*, which inhabits the tropical savannas and grasslands of central Brazil and eastern Paraguay. Here we describe a new karyotype of *Clyomys laticeps* found in populations of Emas National Park, Goiás state, Brazil. The four analyzed specimens had a diploid number (2n) of 32 and a fundamental autosome number (FN) of 54. Cytogenetic data include conventional staining, CBG and GTG-banding. The karyotype presents 12 meta/submetacentric pairs (1 to 12) and 3 pairs of acrocentrics (13 to 15) with gradual decrease in size. The X chromosome is a medium submetacentric and the Y is a medium acrocentric. The semifossorial habits together with habitat specificity could have contributed to the karyological variations found on this genus.

## Introduction

The genus *Clyomys* Thomas, 1916 has long been represented by two living species, namely *Clyomys laticeps* and *Clyomys bishopi* Avila-Pires et Wutke, 1981distributed in tropical savannas and grasslands from circa 100 to 1,100 m elevation in central Brazil and eastern Paraguay (Woods and Kilpatrick 2005). *Clyomys laticeps* would range from the Paraguayan Chaco to the Brazilian States of Minas Gerais and Bahia throughout the Cerrado domain, whereas *Clyomys bishopi* was restricted to the Cerrado enclaves in São Paulo State, Brazil (Avila-Pires and Wutke 1981).Bezerra and Oliveira (2010) have recently reviewed the genus. These authors considered *Clyomys bishopi* a synonym of *Clyomys laticeps* based on quantitative and qualitative characters of skull, phallic morphology, and pelage patterns.

Spiny rats of the genus *Clyomys* present semifossorial habit and can be identified, together with the other semifossorial echimyids genera *Carterodon* Waterhouse, 1848and *Euryzygomatomys* Goeldi, 1901 by a set of morphological characters such as a body covered by spinous pelage, short tail and limbs, and long, powerful claws (Bishop 1974). *Clyomys* differ from those genera by its very conspicuous and hypertrophied auditory bullae (Thomas 1916).

Cytogenetic studies of the genus *Clyomys* reported a diploid number (2n) of 34 chromosomes and fundamental autosome number (FN) 60 or 62. The population from State of São Paulo, Brazil, described by Yonenaga (1975) and by Souza and Yonenaga (1984), showed 2n = 34 and FN = 60, while Svartman (1989) analyzed specimens from Distrito Federal, Brazil, and found the same diploid number, but FN = 62.

The present paper describes a different diploid number for *Clyomys laticeps* from a Central Brazilian sample. We also discuss habitat use and biology of this species and their bearing on the observed intraspecific karyotypic variation.

## Material and methods

**Figure 1.**
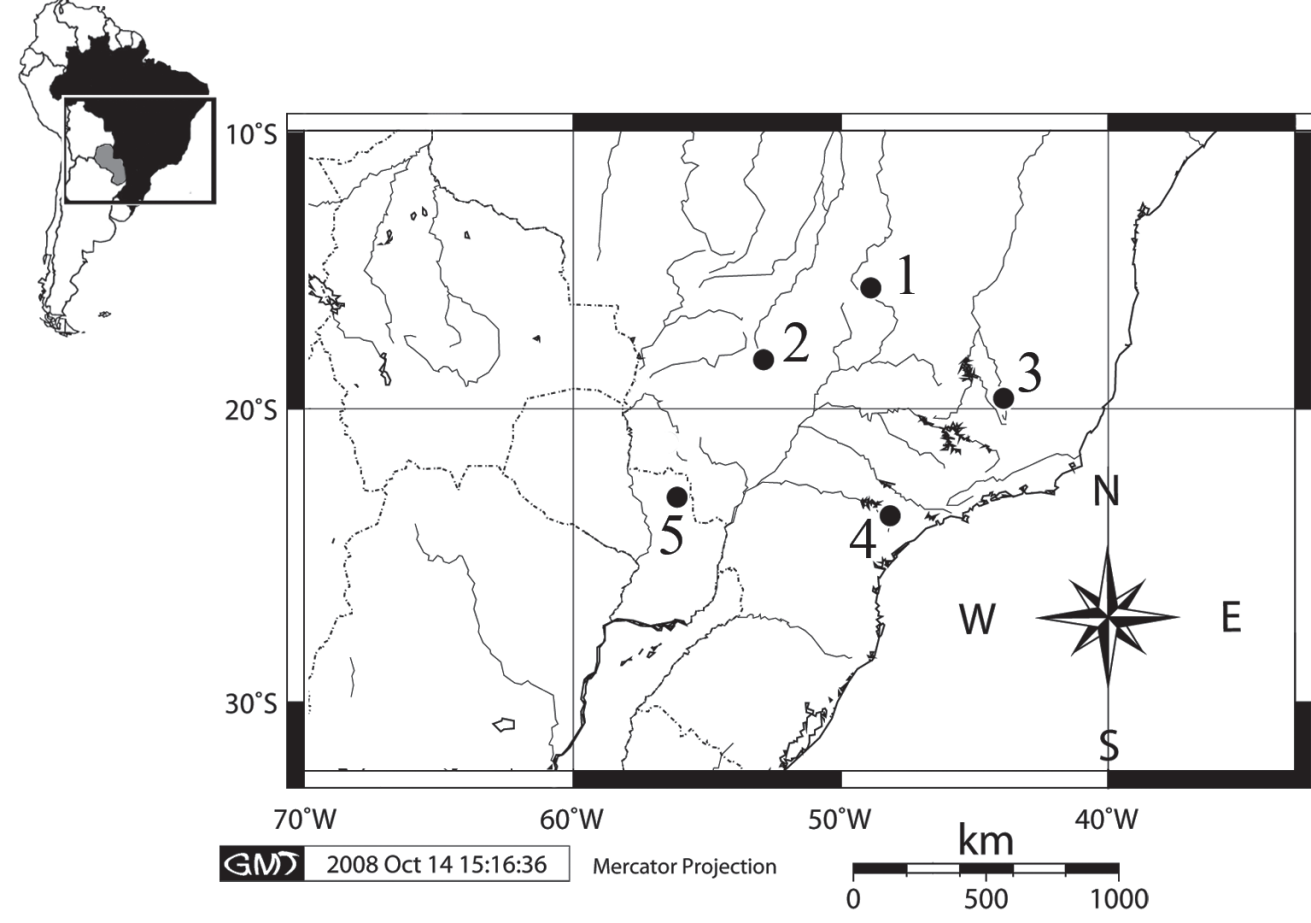
Map of karyotyped populations showing the type localities of *Clyomys laticeps* and its synonyms: Brazil, Distrito Federal, Brasília [**1**] (Svartman 1989); Goiás state, Mineiros, Emas National Park [**2**] (this study); Minas Gerais State, Lagoa Santa [**3**] (type locality of *Clyomys laticeps* – Thomas 1909); São Paulo state, Itapetininga [**4**] (Yonenaga 1975, Souza and Yonenaga 1984; also type locality of *Clyomys bishopi* – Avila-Pires and Wutke 1981); Paraguay, Departamento de San Pedro, Partido de Tacuatí, Acai-Poi [**5**] (type locality of *Clyomys laticeps whartoni* – Moojen 1952). Small map of the South America show the Brazil in black colour and the Paraguay in gray.

Four wild-caught specimens (2 females and 2 males) of *Clyomys laticeps* from Emas National Park (ENP), state of Goiás, Brazil (18°15'50"S, 52°53'33"W) were karyotypically studied (Fig. 1). The vouchers specimens are deposited at the Museu Nacional (MN), Universidade Federal do Rio de Janeiro, and at the Mammal Collection of the Universidade de Brasília (UNB), Brazil: MN 68165 (female), MN 68164 (male), MN 68167 (male), and UNB 2155 (female). The map was generated using the software GMT (2009).

Mitotic metaphase cells were obtained from bone marrow and spleen after *in vivo* colchicine treatment. Mitotic cells were spread onto clean glass slides, air-dried and stored at -20^o^ C until use. Analysis were performed after routine Giemsa staining, CBG-banding (Sumner 1972) and GTG-banding techniques (Seabright 1971).

## Results

**Figure 2.**
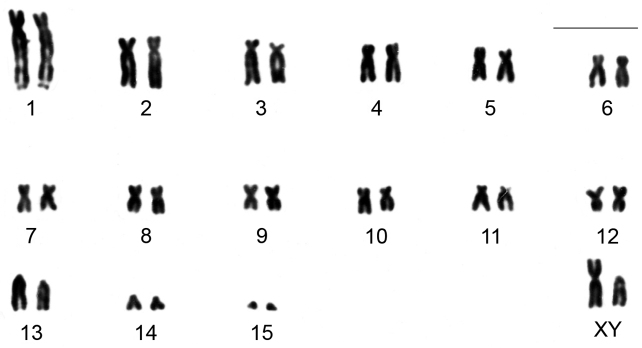
Karyotype of a male of *Clyomys laticeps* (MN 68164) from Emas National Park after conventional staining (2n = 32, FN = 54). Bar = 10 μm.

*Clyomys laticeps* from ENP shows a karyotype with 2n = 32 and FN = 54. The autosome complement comprises 12 biarmed pairs (pair 1 is submetacentric with a distal secondary constriction in the long arm, pairs 2 to 12 are metacentric or submetacentric chromosomes) and three acrocentric pairs (pair 13 a heteromorphic medium acrocentric and pairs 14 and 15 are small acrocentrics). The X chromosome is submetacentric and Y is acrocentric, both morphologically distinguishable after G and/or C banding pattern (see below and Fig. 2).

**Figure 3.**
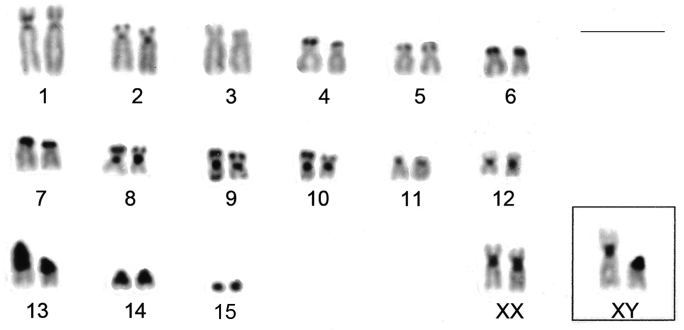
CBG-banded karyotypes of female of *Clyomys laticeps* (UNB 2155) from Emas National Park (2n = 32, FN = 54). Inset: sex chromosomes of a male (MN 68165). Bar = 10 μm.

**Figure 4.**
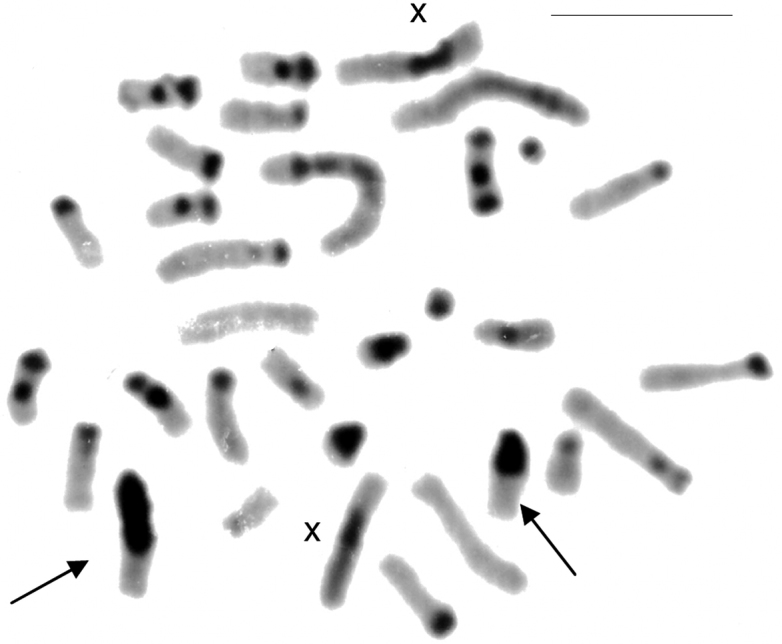
CBG-banded metaphase of a female of *Clyomys laticeps* (UNB 2155) from Emas National Park (2n = 32, FN = 54). The arrows indicate the heteromorphic 13^th^ pair. Bar = 10 μm.

**Figure 5.**
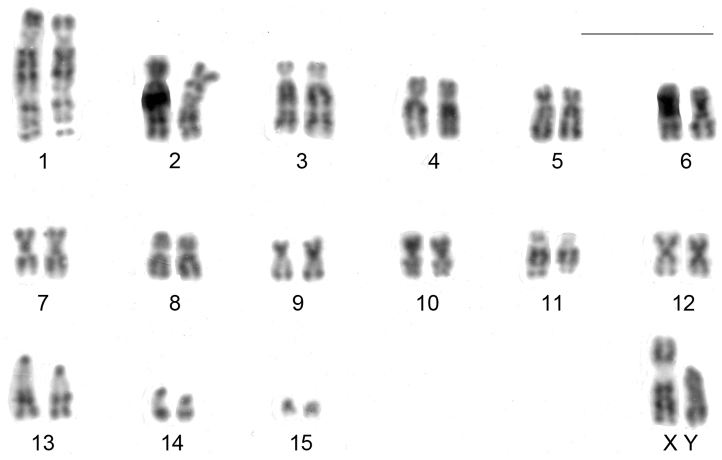
GTG-banded karyotypes of a male of *Clyomys laticeps* (MN 68164) from Emas National Park (2n = 32, FN = 54). Bar = 10 μm.

CBG-banding revealed constitutive heterochromatin at the telomeric regions of some autosomes (pairs 2 and 4–10). Additionally, interstitial bands occurred in the pericentromeric region of pairs 2 and 8–10. The distal secondary constriction in the long arm of pair 1 is C-band negative and the proximal region of both arms shows a small amount of faintly stained constitutive heterochromatin. Pair 3 is completely C-band negative. Pericentromeric positive C-band was present in the pairs 11 and 12, as well as in pairs 13 and 14, which additionally show a large block of heterochromatin in the proximal region of the long arm. The 15^th^ pair is completely heterochromatic. The 13^th^ autosome pair is heteromorphic due to the size of constitutive heterochromatin in all studied specimens (Fig. 2). The X chromosome is identifiable by a large submetacentric with distinctive centromeric heterochromatin and by an unique G-banding pattern characterized by a wide negative G-band at pericentromeric region (Figs 3 and 5). The Y chromosome is an acrocentric similar in size to the smaller acrocentric of the pair 13. It has a conspicuous C-positive band segment in the pericentromeric region and a block at the proximal region of the long arm (Figs 3 and 4). It is readily identifiable only after G-banding since it is G-positive along all its length comparing to the autosome pair 13 (Fig. 5).

## Discussion

The cytogenetic analysis carried out in *Clyomys laticeps* from Emas National Park, Goiás state, Brazil, revealed a new karyotype, with 2n = 32, FN = 54. The specimens of *Clyomys laticeps* described in the literature from São Paulo state and from Distrito Federal, respectively, shared very similar 2n = 34 karyotypes with a minor difference only in fundamental autosome number (60/62). Specimens from Itapetininga, São Paulo state, the type locality of *Clyomys bishopi*, also showed 2n = 34 and FN = 60 (Yonenaga 1975, Souza and Yonenaga 1984). The autosomes from São Paulo state populations were composed by one large acrocentric pair (pair 1), 13 pairs of metacentrics or submetacentrics and 2 small pairs of acrocentric chromosomes (15 and 16). The specimens from Distrito Federal showed a karyotype with 2n = 34 and FN = 62 (Svartman 1989), with 14 pairs of metacentrics or submetacentrics, one pair of subtelocentric and 1 pair of acrocentric chromosomes. All spiny rats of the family Echimyidae present only one chromosome pair with a large secondary constriction and the karyotypes described for the genus *Clyomys* show this characteristic (Leal-Mesquita et al. 1992, Souza and Yonenaga 1984, Svartman 1989, present study).

The cytogenetic distinction between 2n = 34 karyotypes from São Paulo (with FN = 60) and Distrito Federal (FN = 62) could be the result of a rearrangement such as a pericentric inversion on one pair of chromosomes. The difference in diploid number between the karyotypes of *Clyomys laticeps* with 2n = 32 and 2n = 34, in the other hand, might mostly be related to Robertsonian rearrangements (fusion/fission events).

The origin of the diploid number differences is probably the result of a series of complex rearrangements. The karyotypes with 2n = 34 (from São Paulo specimens) are composed by two small pairs of acrocentric chromosomes (15 and 16) while in specimens with 2n = 32 from ENP there are three pairs of small acrocentrics (13, 14 and 15). There is correspondence between the C-band pattern between the pairs 15 and 16 of *Clyomys laticeps* from São Paulo stateand the pairs 14 and 15 from the ones of ENP. Therefore, the karyotypes with the smallest diploid number present an additional small acrocentric pair (13). Moreover, the first pair of chromosomes in the karyotype of São Paulo specimens (2n = 34) is a large acrocentric with a small quantity of heterochromatin in the pericentromeric region, while the first pair in ENP specimens (2n = 32) is a submetacentric with a small amount of faintly stained constitutive heterochromatin in the proximal region of both arms. The X chromosome is also morphologically distinct between the karyotypes analyzed, being an average sized acrocentric in São Paulospecimensand a submetacentric in specimens from ENP. The constitutive heterochromatin in the X chromosome of 2n = 34 karyotypes is located in the pericentromeric region and in the proximal region of the long arm, while in the X chromosome of karyotypes with 2n = 32 a pericentromeric heterochromatic band is present. Thus, events such as addition/deletion of heterochromatin and pericentric inversions associated with centric fission/fusion (Robertsonian rearrangements) might have happened in the evolutionary differentiation of the karyotypes of these two populations.

Cytogenetic variability in fossorial and semifossorial rodents has been widely reported in the literature (e.g., Hafner et al. 1987, Nevo et al. 1990, Sulentich et al. 1991, Garcia et al. 2000), often inferred as a consequence of population structuring imposed by the specialized fossorious habit (Reig et al. 1990). Spiny rats of the species C. *laticeps* have semifossorial habits (Amante 1975, Carvalho and Bueno 1975, Lacher and Alho 1989) and are the most phylopatric individuals in non-volant small mammal communities (Vieira 1997, Bezerra and Oliveira 2010), suggesting that this rodent is a habitat-specialist that needs soils with a soft structure that permits easily burrowing (Bezerra and Oliveira 2010).

The distinct diploid number shown by *Clyomys* populations (Yonenaga 1975, Souza and Yonenaga 1984, Svartman 1989, this study) could constitute evidence of speciation if one uses a biological species concept. However, additional cytogenetic data, like *in situ* hybridization (FISH) of telomeric sequences, Zoo-FISH, and the chromosomal characterization of other *Clyomys* populations are still necessary to provide us a better comprehension of the mechanisms involved in the chromosome differentiation and, consequently, in the speciation of this genus.
